# Effect of LRRK2 G2385R Variant on Subthalamic Deep Brain Stimulation Efficacy in Parkinson's Disease in a Han Chinese Population

**DOI:** 10.3389/fneur.2019.01231

**Published:** 2019-11-22

**Authors:** Si Chen, Hong Liu, Qian-qian Wu, Shu-jun Xu, Wei-guo Li, Teng Chen, Chao Li, Xiang-yu Ma, Shuo Xu, Yi-ming Liu

**Affiliations:** ^1^Department of Neurosurgery, Qilu Hospital of Shandong University, Jinan, China; ^2^Department of Neurology, People's Hospital of Liaocheng, Liaocheng, China; ^3^Department of Neurology, Qilu Hospital of Shandong University, Jinan, China

**Keywords:** Parkinson's disease, deep brain stimulation, Han Chinese, *LRRK2*, STN-DBS surgery, G2385R

## Abstract

Deep brain stimulation (DBS) of the subthalamic nucleus (STN) is an effective treatment for advanced Parkinson's disease (PD). The G2385R variant of *LRRK2* is a risk factor for PD in Han Chinese individuals. We retrospectively compared the clinical outcomes of STN-DBS surgery between PD Han Chinese *G2385R* variant carriers and non-carriers. Fifty-seven PD patients with bilateral STN-DBS were enrolled, including 8 G2385R+ variant carriers (G2385R+ group) and 49 non-carriers (G2385R– group). Clinical data included Unified Parkinson's Disease Rating Scale (UPDRS) parts I to IV, levodopa equivalent daily dose (LEDD), Mini-Mental State Examination Scale (MMSE) score, and Hamilton Depression Rating Scale (HAMD) score measured prior to DBS and 12 months post-DBS. DBS settings were also recorded. All PD patients benefited from STN-DBS surgery. There were no statistical differences between the two groups in terms of motor function, daily living activities, and LEDD reductions at 12 months post-DBS. The rigidity of the post-surgical G2385R+ group was significantly improved compared with that of the G2385R– group (*P* = *0.045*). Post-surgical voltage in the G2385R+ group was significantly higher than that in the G2385R– group (*P* = *0.033*). STN-DBS outcomes were not influenced by the *LRRK2* G2385R variant in Han Chinese patients.

## Introduction

Parkinson's disease (PD) is a common and chronic neurodegenerative disorder characterized clinically by movement impairments including resting tremor, bradykinesia, rigidity, and postural instability, as well as a variety of non-motor symptoms. The pathogenesis of PD remains unclear. Aging, genetics, and environmental factors have been identified in idiopathic forms of PD. Mutations in the leucine-rich repeat kinase 2 (*LRRK2*) gene are the most common mutations in sporadic and familial PD. Of these, G2385R and R1628P are the two most common LRRK2 variants in Asian patients with PD (1–3). In particular, the G2385R variant increases the risk of PD in the Han Chinese population by nearly 2-fold (4). The LRRK2 protein is integrated with GTP and protein kinase enzymes and is widely expressed in various tissues and involved in central nervous system functioning, including in the nigrostriatum and caudate nucleus (5). G2385R is located on the C-terminal of the LRRK2's WD40 domain, which is involved in the interaction of LRRK2 with other proteins (1).

Deep brain stimulation (DBS) of the subthalamic nucleus (STN) is widely used in the clinical treatment of advanced PD (6, 7). Critically, the efficacy of DBS surgery varies based on the population of PD patients treated. Patients who meet the following criteria often achieve satisfactory surgical results (8–10), including good responsivity to levodopa, a shorter disease duration, younger age at onset, levodopa-responsive axial motor symptoms, and normal cognition and mental status. However, whether genetic factors influence STN-DBS outcomes remains unclear (11, 12). Previous studies and case reports have found that PD patients with various genetic mutations have differing treatment responses to DBS. However, no existing reports have focused on the outcomes of bilateral STN-DBS in Han Chinese PD patients positive for the G2385R variant. To test this, we retrospectively compared clinical outcomes of STN-DBS surgery between G2385R variant carriers and non-carriers in Han Chinese PD patients.

## Materials and Methods

### Participants

A total of 95 PD patients of Han nationality who underwent bilateral STN-DBS surgery in the Department of Neurosurgery at Qilu Hospital of Shandong University between January 2015 and December 2017 were included. All patients were diagnosed with PD by movement disorder specialists in the Department of Neurology at Qilu Hospital of Shandong University, using the United Kingdom Parkinson's Disease Society Brain Bank criteria and met the criteria for DBS surgery, as described previously (6, 13). Among them, 57 patients with specific clinical data and DNA samples agreed to participate in this study. Patients with family history of PD were excluded.

### Genetic Analysis

Peripheral blood samples were collected from all participants for DNA extraction. DNA was extracted from peripheral blood leukocytes by standard procedures. Genomic DNA was amplified by polymerase chain reaction (PCR). PCR primers for G2385R and R1628P variants were used, as reported previously (3, 4). PCR products were sequenced by an ABI 3730xl DNA analyzer (Applied Biosystems, Inc., Foster City, CA, USA) and data were analyzed using ChromasPro software (Technelysium, South Brisbane, Australia). G2385R+/R1628P– patients were assigned to the positive G2385R group (G2385R+ group) and G2385R–/R1628P– patients were assigned to the negative G2385R group (G2385R– group). Written, informed consent was obtained from all participants and the study was approved by the Ethics Committee of Qilu Hospital of Shandong University [Approval number: KYLL-2017(KS)270].

### Clinical Assessments

Clinical data from 57 PD patients were collected at a pre-surgical timepoint (baseline), and then again 12 months after surgery. However, a sub-set of these volunteers were also followed up at later time-points including a 24 month post-surgery follow-up (*n* = 9) and a 36 month post-surgery follow-up (*n* = 5). Clinical data included the Mini-Mental State Examination Scale (MMSE), the Hamilton Depression Rating Scale (HAMD), the Unified Parkinson's Disease Rating Scale (UPDRS-I: Cognition, Behavior, and Emotion; UPDRS-II: Daily Activity; UPDRS-III: Motor Function; UPDRS-IV: Levodopa-induced Motor Fluctuations and Dyskinesia), and levodopa equivalent daily dose (LEDD) (14). In addition, the following specific subscores from the off-medication UPDRS-III were also included: tremor (UPDRS-III questions 20 and 21), rigidity (UPDRS-III question 22), bradykinesia (UPDRS-III questions 23–26), and axial symptoms (UPDRS III questions 27–30). Post-DBS stimulation settings were also recorded.

The primary outcomes were changes in off-medication UPDRS-II scores and UPDRS-III scores and decreases in LEDD from the pre-DBS baseline to 12 months post-DBS. These were compared between groups. Secondary outcomes included the UPDRS-I scores and off-medication UPDRS-III subscores, UPDRS-IV scores, MMSE and HAMD, and post-DBS stimulation settings.

### Statistical Analyses

Data were analyzed using SPSS 22.0 (IBM Corporation, Armonk, NY, USA). If normally distributed, measurements were expressed as means ± standard deviations. Otherwise, percentiles (P25–P75) were used for non-normal distributions. For categorical data, data were described in the form of frequencies (percentages). Between-group pre-surgical measurements were compared using *t*-tests or non-parametric tests, while categorical data were compared with chi-squared or Fisher's exact probability tests for between-group comparisons. A linear mixed model was used for post-surgical group comparisons. Paired *t*-tests or Kruskal-Wallis non-parametric tests were used to compare preoperative and postoperative indexes. The chi-square test was used to compare stimulation settings. *P* < 0.05 was used to detect statistical significance.

## Results

### Baseline Demographics and Clinical Characteristics

Fifty-seven PD patients with bilateral STN-DBS (20 with units produced by Medtronic, USA, 37 by PINS Medical, China) were enrolled, including 8 patients in the G2385R+ group and 49 patients in the G2385R– group. The G2385R+ group accounted for 14% of the total sample size. Patients' basic pre-surgical demographics and clinical data are shown in Table 1. There were no significant differences in age, gender, age at onset, disease duration, motor symptoms, levodopa responsiveness, or Hoehn and Yahr Scale between the groups.

**Table 1:** Baseline G2385R+ and G2385R− group characteristics.

	**G2385R+ (*n =* 8)**	**G2385R– (*n =* 49)**	***P***
Age at surgery (years)	62.38 ± 9.84	61.29 ± 8.13	0.734
Male/Female	4/4	23/26	0.872
Age at PD onset (years)	52.13 ± 7.55	51.80 ± 8.51	0.919
Disease duration (years)	10.25 ± 3.73	9.76 ± 4.17	0.754
UPDRS III (off medication)	43.38 ± 11.51	49.00 ± 11.62	0.209
UPDRS III (on medication)	15.88 ± 8.34	20.55 ± 10.43	0.234
Levodopa responsiveness (%)	61.15 ± 18.48	58.81 ± 18.21	0.738
Hoehn and Yahr Scale	2.94 ± 0.78	2.90 ± 0.67	0.880

*Data shown as means ± standard deviations*.

*UPDRS, Unified Parkinson's disease Rating Scale*.

### Group-Wise Comparisons

There were no significant differences in UPDRS parts I to IV, LEDD, MMSE, and HAMD between the two groups in pre-surgical baseline characteristics (Table 2).

**Table 2:** Preoperative and postoperative clinical characteristics of G2385R– and G2385R+ group participants with bilateral STN-DBS.

	**Pre-surgical baseline**			**12-month post-surgery**		
	**G2385R– (*n =* 49)**	**G2385R+ (*n =* 8)**	***P***	**G2385R– (*n =* 49)**	**G2385R+ (*n =* 8)**	***P***
UPDRS I	4.14 ± 2.25	5.13 ± 1.73	0.244	2.51 ± 1.75	2.88 ± 1.96	0.790
UPDRS II	23.02 ± 7.19	25.50 ± 7.50	0.372	14.76 ± 5.44	13.13 ± 5.46	0.172
UPDRS II improvement, %	–	–	–	35.48 (18.47–47.27)	36.95 (33.33–66.31)	0.183
UPDRS III	Off medication			Off medication/Stimulation on		
Total scores	49.00 ± 11.62	43.38 ± 11.51	0.209	29.69 ± 9.95	20.50 ± 7.89	0.193
Tremor	6.00 (0.50–12.00)	3.50 (0.00–6.50)	0.128	0.00 (0.00–1.50)	0.00 (0.00–1.75)	0.401
Rigidity	11.20 ± 3.73	8.50 ± 2.51	0.054	6.53 ± 2.69	4.13 ± 1.96	0.045
Akinesia	15.98 ± 5.47	15.88 ± 5.17	0.960	10.12 ± 4.62	7.25 ± 2.92	0.094
Axial symptoms	6.43 ± 2.26	6.75 ± 2.19	0.321	4.41 ± 2.18	3.75 ± 2.61	0.352
UPDRS III improvement, %	–	–	–	45.45 (28.01–58.51)	46.08 (26.88–64.92)	0.778
UPDRS IV						
Dyskinesia	1.00 (0.00–2.00)	2.00 (0.00–3.00)	0.435	0.00 (0.00–1.50)	0.00 (0.00–1.50)	0.651
Motor fluctuations	3.82 ± 1.17	3.88 ± 1.13	0.895	3.00 (1.00–4.00)	2.50 (1.00–3.00)	0.453
LEDD (mg/d)	885.96 ± 381.34	1041.41 ± 298.15	0.278	544.23 ± 257.01	600.59 ± 198.95	0.823
LEDD reduction, %	–	–	–	37.50 (24.49–52.50)	41.95 (23.44–52.03)	0.727
MMSE	25.51 ± 4.29	26.63 ± 2.26	0.478	25.65 ± 4.15	26.75 ± 2.87	0.858
HAMD	15.49 ± 7.10	19.75 ± 9.16	0.136	10.00 (5.00–15.00)	12.50 (3.75–21.50)	0.761

*Described by mean ± standard deviation (normal distribution) and P50 (P25-P75) (skewed distribution)*.

*UPDRS, Unified Parkinson's Disease Rating Scale; LEDD, levodopa equivalent daily dose; MMSE, Mini-Mental State Examination Scale; HAMD, Hamilton Depression Rating Scale*.

A linear mixed model was used to compare the influence of the G2385R variant on the effects of STN-DBS (15). We found no statistical differences between the two groups in the three primary outcomes at 12 months post-DBS. Among the secondary outcomes, post-surgery rigidity in the G2385R+ group was significantly improved compared to the G2385R– group levels (*P* = 0.045; Table 2).

### Baseline vs. Post-surgery

In both groups, UPDRS-II and III off-medication scores improved remarkably 12 months post-surgery. UPDRS-II scores in the G2385R+ and G2385R– groups improved by 36.95 and 35.48%, respectively. UPDRS-III score percent changes were 46.08 and 45.45%, respectively. LEDD reductions were 41.95 and 37.50% in the two groups, respectively (Table 2).

Table 3 shows the comparisons of the postoperative scores to preoperative scores in both groups. While the G2385R– group improved on all scores except for the MMSE, the G2385R+ group improved on all scores except for the MMSE, HAMD, tremor, axial symptom, dyskinesias, and motor fluctuations.

**Table 3:** Comparison of outcomes between preoperative baseline and 12 months post-operation in the G2385R− and G2385R+ groups.

	**G2385R– (*n =* 49)**			**G2385R+(*n =* 8)**		
	**Pre-surgical baseline**	**12-month post-surgery**	***P***	**Pre-surgical baseline**	**12-month post-surgery**	***P***
UPDRS I	4.14 ± 2.25	2.51 ± 1.75	<0.001	5.13 ± 1.73	2.88 ± 1.96	0.029
UPDRS II	23.02 ± 7.19	14.76 ± 5.44	<0.001	25.50 ± 7.50	13.13 ± 5.46	0.002
UPDRS III						
Total scores	49.00 ± 11.62	29.69 ± 9.95	<0.001	43.38 ± 11.51	20.50 ± 7.89	0.004
Tremor	6.00 (0.50–12.00)	0.00 (0.00–1.50)	<0.001	3.50 (0.00–6.50)	0.00 (0.00–1.75)	0.115
Rigidity	11.20 ± 3.73	6.53 ± 2.69	<0.001	8.50 ± 2.51	4.13 ± 1.96	0.009
Akinesia	15.98 ± 5.47	10.12 ± 4.62	<0.001	15.88 ± 5.17	7.25 ± 2.92	0.006
Axial symptoms	6.43 ± 2.26	4.41 ± 2.18	<0.001	6.75 ± 2.19	3.75 ± 2.61	0.060
UPDRS IV						
Dyskinesia	1.00 (0.00–2.00)	0.00 (0.00–1.50)	0.038	2.00 (0.00–3.00)	0.00 (0.00–1.50)	0.121
Motor fluctuations	3.82 ± 1.17	3.00 (1.00–4.00)	<0.001	3.88 ± 1.13	2.50 (1.00–3.00)	0.084
LEDD (mg/d)	885.96 ± 381.34	544.23 ± 257.01	<0.001	1041.41 ± 298.15	600.59 ± 198.95	0.001
MMSE	25.51 ± 4.29	25.65 ± 4.15	0.621	26.63 ± 2.26	26.75 ± 2.87	0.785
HAMD	15.49 ± 7.10	10.00 (5.00–15.00)	<0.001	19.75 ± 9.16	12.50 (3.75–21.50)	0.107

*Described by mean ± standard deviation (normal distribution) and P50 (P25-P75) (skewed distribution)*.

*UPDRS, Unified Parkinson's Disease Rating Scale; LEDD, levodopa equivalent daily dose; MMSE, Mini-Mental State Examination Scale; HAMD, Hamilton Depression Rating Scale*.

The comparison of 9 PD patients between baseline and the 12 and 24 month follow-ups are described in Supplementary Table 1. At two follow-up sites, UPDRS-II and UPDRS-III (including subscore rigidity) scores were decreased; whereas LEDD scores were significantly reduced relative to baseline. Supplementary Table 2 shows the changes of 5 PD patients in a 36 month post-surgery follow-up relative to baseline. Except for the significant decrease in UPDRS-III scores, there were no significant differences in other scores.

### Stimulation Settings

The stimulation settings for the two groups are shown in Table 4. Eight patients in the G2385R+ group received 16 sets of parameter settings and 49 patients in the G2385R– group obtained 98 sets of parameter settings. There were no statistically significant differences in proportional distributions of monopolar stimulation, bipolar stimulation, double monopolar stimulation, and interleaved stimulations between the two groups. The postoperative voltage used in PD patients in the G2385R+ group was significantly higher than that used in G2385R– group patients (*P* = 0.033). However, there was no significant difference in pulse width or frequency between the two groups.

**Table 4:** Stimulation settings.

	**G2385R– (*n =* 98)**	**G2385R+ (*n =* 16)**	***P***
Monopolar stimulation, *n* (%)	61 (62.24)	7 (43.75)	0.162
Bipolar stimulation, *n* (%)	1 (1.02)	0 (0)	1.000
Double monopolar stimulation, *n* (%)	24 (24.49)	6 (37.5)	0.430
Interleaved stimulation, *n* (%)	12 (12.24)	3 (18.75)	0.753
Voltage (V)	2.42 ± 0.41	2.64 ± 0.35	0.033
Pulse width (μs)	73.64 ± 14.12	76.84 ± 14.93	0.224
Frequency (Hz)	151.95 ± 37.83	140.26 ± 42.64	0.367

*Data shown as mean ± standard deviation*.

## Discussion

In the present study, 8 G2385R+ PD patients and 49 G2385R– PD patients with bilateral STN-DBS who were similar in ethnicity (Han Chinese), age, gender, age at PD onset, age at DBS surgery, duration of disease, and other clinical features were included. The symptoms of patients in both groups improved after bilateral STN-DBS. We found no significant differences in motor function, activities of daily living, and LEDD reductions between the G2385R+ and G2385R– groups post-DBS, indicating that the G2385R variant did not significantly influence the effects of DBS on PD symptoms in this population.

LRRK2 gene was first described in a large Japanese family with autosomal dominant PD (16). The gene was subsequently confirmed in several European families. However, the phenotype of LRRK2-related PD is difficult to distinguish from idiopathic PD. The clinical manifestations of LRRK2-related PD have been described as being more benign than those shown in idiopathic PD, where motor symptoms (disease severity, rate of progression, occurrence of falls, and dyskinesia) and non-motor symptoms (cognition and olfaction) are less severe (17), whereas familial PD caused by LRRK2 have been described as being similar to idiopathic PD (18). Unlike idiopathic PD patients, the clinical features of G2385R-related PD remain unclear. When compared with non-carriers, carriers of the G2385R variant experience a longer disease duration (19), an earlier age of onset (4), a higher proportion of postural instability and gait disorder (PIGD) and motor fluctuations, and higher LEDD as compared to non-carriers (20). However, there are also clinical studies suggesting that G2385R carriers have clinical features similar to idiopathic PD patients (21). A recent meta-analysis reported an association between the G2385R variant in LRRK2 and idiopathic PD phenotypes, demonstrating no significant difference in gender distribution, age at onset, initial symptoms, motor symptoms, depression, LEDD, or motor complications (22).

The efficacy of DBS in patients carrying the LRRK2 variant have been investigated in several studies. These studies have mostly focused on the G2019S variant (15, 23–26), which differs from the G2385R variant in its geographical distribution and clinical characteristics. As mentioned above, G2385R variants are more often found in patients from East Asia (7.3–11.8%) (27). According to two previous studies, the prevalence of this variant in Han Chinese PD patients was 11.8 and 8.3%, respectively (4, 28). While G2019S variants are most prevalent in sporadic PD patients of Westerners, Ashkenazi Jewish individuals (10–15%), and North African Arabic individuals (~40%) (15). A recent study showed that the Movement Disorder Society-Sponsored Revision of the Unified Parkinson's Disease Rating Scale (MDS-UPDRS) part II and III scores and the frequency of motor fluctuations among G2385R variant carriers were higher than among either G2019S variant carriers or idiopathic PD patients in a sample of 516 G2019S variant carriers, 199 G2385R variant carriers and 790 patients with idiopathic PD (29). The incidence of the PIGD phenotype among G2385R and G2019S variant carriers was also higher than that among idiopathic PD patients. Previous studies have shown that patients with the G2019S variant respond better to DBS than non-variant carriers (24), but the effect of the G2385R variant on the efficacy of DBS remains unclear. The present study addressed this gap by revealing that the G2385R variant had no significant influence on primary outcomes in our patient population. According to the analyzed outcomes, it slightly improved the secondary outcome of rigidity (*P* = 0.045), whereas the difference of rigidity score at the pre-surgical baseline showed a slightly lower value (*P* = 0.054). Moreover, the improvement of rigidity, which is one of the symptoms of PD, may not convey valuable information for the impact of G2385R variant on DBS efficacy.

After analyzing the postoperative stimulation settings, we found that the stimulation voltage used in the G2385R+ group was significantly higher than that used in the G2385R– group. However, there were no significant differences in frequency, pulse width, or parameter settings between the two groups. All patients were programmed with constant pressure. As an important measure of stimulation energy, the total electrical energy delivered (TEED) was calculated using the following formula: [(voltage^2^× pulse × width × frequency)/impedance] (30). Due to a lack of impedance data, TEED could not be calculated for further comparison. Stimulation settings are key to the efficacy of DBS (31), with differences in postoperative parameter settings potentially causing differential improvements in motor symptoms. Voltage is the most prominent factor in improving motor symptoms in PD with DBS (32). In the present study, differences in voltage between the two groups may have influenced the evaluation of postoperative motor symptoms. Furthermore, while postoperative stimulation settings and DBS-related efficacy are rarely reported in PD-by-genotype studies, these factors may be relevant confounds (11).

The impact of STN-DBS on cognitive function in PD patients has been controversial. Studies have reported that 36% of PD patients experience some cognitive decline 12 months after STN-DBS as compared to control patients (33). In the present study, we found no significant changes in MMSE between the two groups at 12 months post-surgery. However, the most consistent results from long-term follow-ups assessing cognitive changes after STN-DBS include decreased verbal fluency and executive functioning, symptoms which are consistent with the progression of parkinsonian dementia (34). Therefore, our use of the MMSE alone in the present study did not allow us to unequivocally conclude that cognitive function remained unaffected among patients in the present study. Future work should address verbal fluency and executive functioning more directly.

Several limitations of the present study should be acknowledged. First, the sample size of G2385R carriers included here was small. As such, we cannot exclude the possibility that our sample may have been statistically underpowered. Although the two groups were not matched by sample size, gender, age at onset, or disease duration, there were no significant differences in these factors or in motor symptoms or Hoehn and Yahr Scale pre-surgery. Moreover, since the total number of participants was relatively small, the frequency of the G2385R variant seems to be slightly higher than expected. A larger population of G2385R variant carriers is therefore needed to draw more conclusive results.

An additional limitation of the present study was its retrospective design. Due to gaps in the clinical data, many non-motor symptoms associated with PD, such as sleep disorder, fatigue, or dysfunction of the autonomic nervous system, were not specifically analyzed here. Non-motor symptoms are very important clinical features of PD and it is possible that the G2385R variant specifically affects these features of PD following DBS surgery. Future studies are needed to address this.

Finally, G2385R seems to be a risk factor for PD rather than a pathogenic, causative mutation. One hypothesis is that interactions with other genes or environmental factors may influence the penetrance and expression of G2385R mutation, thereby increasing the risk for developing PD (35). However, a recent study demonstrated that the G2385R variant altered the strength and quality of LRRK2 interactions and increased the rate of synaptic vesicle fusion. Given this, the G2385R variant may behave like a loss-of-function mutation (36).

In summary, understanding patients' genetic backgrounds is critical to understanding the clinical heterogeneity of PD and their varying responses to treatment. Given this, genetics should be carefully considered in the preoperative evaluation of PD patients and candidates for DBS. The results obtained in the present study help to clarify the potential influence of the G2385R variant on DBS outcomes in Parkinson's disease patients with a Han Chinese background. The results of the present study provide some critical guidance on the preoperative evaluation of these patients.

## Data Availability Statement

The raw data supporting the conclusions of this manuscript will be made available by the authors, without undue reservation, to any qualified researcher.

## Ethics Statement

The studies involving human participants were reviewed and approved by Ethics Committee of Qilu Hospital of Shandong University. The patients/participants provided their written informed consent to participate in this study.

## Author Contributions

ShuX, YL, and SC contributed to conception and design of the study. ShuX, WL, TC, CL, XM, and ShuoX performed the surgery. SC and QW performed the statistical analysis and drafted the manuscript. All authors contributed to manuscript revision, read, and approved the submitted version.

### Conflict of Interest

The authors declare that the research was conducted in the absence of any commercial or financial relationships that could be construed as a potential conflict of interest.
