# Supplementary material for: Effect of LRRK2 G2385R Variant on Subthalamic Deep Brain Stimulation Efficacy in Parkinson's Disease in a Han Chinese Population
Source: Front Neurol. 2019 Nov 22;10:1231. doi: 10.3389/fneur.2019.01231 (PMC6884002; doi:10.3389/fneur.2019.01231)
Supplement: Supplementary file 1 [file Data_Sheet_1.doc]

|  | 24-month follow-up (n = 9) | | | P | |
| --- | --- | --- | --- | --- | --- |
|  | Pre-surgical baseline | 12-month post-surgery | 24-month post-surgery | Baseline vs 12-month post-surgery | Baseline vs 24-month post-surgery |
| UPDRS I | 3.56 ± 1.74 | 3.33 ± 1.50 | 2.78 ± 1.72 | 0.594 | 0.065 |
| UPDRS II | 23.67 ± 6.48 | 17.78 ± 6.16 | 18.00 ± 6.22 | 0.006 | < 0.001 |
| UPDRS III | | | | | |
| Total scores | 42.00 ± 16.70 | 28.56 ± 9.46 | 29.89 ± 9.21 | 0.023 | 0.017 |
| Tremor | 4.00 (2.50 -4.50) | 4.00 (3.00 -4.00) | 4.00 (3.00 -4.00) | 0.914 | 0.334 |
| Rigidity | 10.22 ± 3.77 | 6.67 ± 2.35 | 7.56 ± 1.94 | 0.037 | 0.029 |
| Akinesia | 14.89 ± 7.10 | 11.11 ± 4.88 | 10.56 ± 4.30 | 0.165 | 0.067 |
| Axial symptoms | 6.56 ± 3.13 | 5.22 ± 1.79 | 5.89 ± 2.20 | 0.096 | 0.242 |
| UPDRS IV | | | | | |
| Dyskinesia | 0.00 (0.00 -1.00) | 0.00 (0.00 -1.00) | 0.00 (0.00 -2.00) | 0.317 | 0.317 |
| Motor fluctuations | 3.44 ± 1.33 | 3.56 ± 0.53 | 3.78 ± 0.67 | 0.824 | 0.397 |
| LEDD (mg/d) | 878.47 ± 276.12 | 595.67 ± 198.10 | 579.86 ± 184.45 | 0.002 | 0.004 |
| MMSE | 25.11 ± 6.23 | 25.11 ± 5.99 | 25.11 ± 5.84 | 1.000 | 1.000 |
| HAMD | 16.33 ± 6.65 | 17.00 ± 8.90 | 15.56 ± 7.52 | 0.674 | 0.584 |

Supplementary Table 1. Comparison of outcome between baseline and 12- and 24-month post-operative follow-ups

Described by mean ± standard deviation (normal distribution) and P50 (P25-P75) (skewed distribution).

UPDRS: Unified Parkinson’s Disease Rating Scale; LEDD: levodopa equivalent daily dose; MMSE: Mini-Mental State Examination Scale; HAMD: Hamilton Depression Rating Scale.

Supplementary Table 2. Comparison of outcomes between baseline and 36-month post-surgery follow-up

|  | 36-month follow-up (n = 5) | | P |
| --- | --- | --- | --- |
|  | Pre-surgical baseline | 36-month post-surgery |
| UPDRS I | 4.20 ± 1.10 | 3.40 ± 1.34 | 0.294 |
| UPDRS II | 24.00 ± 8.54 | 19.00 ± 3.74 | 0.160 |
| UPDRS III | | | |
| Total scores | 37.60 ± 16.62 | 28.20 ± 10.69 | 0.049 |
| Tremor | 4.00 (3.50-5.00) | 3.00 (1.50 -4.50) | 0.098 |
| Rigidity | 10.60 ± 3.65 | 6.80 ± 2.68 | 0.056 |
| Akinesia | 14.40 ± 8.68 | 10.00 ± 4.12 | 0.106 |
| Axial symptoms | 6.40 ± 3.21 | 6.2 ± 2.49 | 0.799 |
| UPDRS IV | | | |
| Dyskinesia | 0.00 (0.00 -2.00) | 0.00 (0.00 - 2.00) | 1.000 |
| Motor fluctuations | 3.00 ± 1.58 | 4.20 ± 0.84 | 0.109 |
| LEDD (mg/d) | 838.75 ± 312.82 | 654.00 ± 172.66 | 0.138 |
| MMSE | 28.00 ± 2.00 | 27.2 ± 3.56 | 0.456 |
| HAMD | 15.40 ± 6.91 | 17.2 ±8.35 | 0.353 |

Described by mean ± standard deviation (normal distribution) and P50 (P25-P75) (skewed distribution).

UPDRS: Unified Parkinson’s Disease Rating Scale; LEDD: levodopa equivalent daily dose; MMSE: Mini-Mental State Examination Scale; HAMD: Hamilton Depression Rating Scale.
